# PD40 Economic Impact Of Cutaneous Leishmaniasis From The Patient Perspective: An Analysis Of A Reference Center In Brazil

**DOI:** 10.1017/S0266462325101736

**Published:** 2025-12-29

**Authors:** Sarah Silva, Janaína Carvalho, Talia Assis, Endi Lanza Galvão, Gláucia Cota

## Abstract

**Introduction:**

Cutaneous leishmaniasis (CL) is a neglected endemic disease in several states in Brazil. The direct costs of CL treatment from the patient’s perspective can be significant, as can the indirect costs, which include the various non-medical expenses and loss of income incurred during treatment. The study objective was to identify the direct and indirect costs during the treatment of cutaneous leishmaniasis from the patient perspective.

**Methods:**

A cross-sectional study was conducted at the René Rachou Institute/Fiocruz between April 2022 and April 2023 and included patients with a confirmed diagnosis of CL. Participants were interviewed during the treatment period to assess direct medical and non-medical costs as well as indirect costs associated with treatment. Direct costs were estimated using the micro-costing approach and indirect costs were estimated using the human capital method. Descriptive analyses and hypothesis tests were performed for associations between costs and sociodemographic and clinical variables, with a statistical significance level of five percent. The study had ethical approval (CAAE 28929220.0.0000.5091).

**Results:**

The study included 68 patients, predominantly male (77.9%) with an average age of 53 years. CL was the most common clinical form (76.4%), with new cases accounting for 79.4 percent of participants. Direct costs per treatment cycle averaged USD117.36, resulting from transportation, food, and medical examinations. Indirect costs from lost workdays amounted to USD9,936.58, with an average of USD160.12 per patient. Catastrophic expenditure (>10% of monthly income) was observed in 41.9 percent of families. This was significantly associated with direct cost, bacterial infection, and sociodemographic factors such as sex, age, and distance traveled.

**Conclusions:**

This study confirmed that treatment for CL, although free in the Brazilian health system, generates significant direct and indirect costs for patients. The highest costs are related to travel and accommodation resulting from the centralized healthcare model, where treatment is not available close to where patients live.

